# Obesity and insulin resistance are significant predictors of serum leptin levels

**DOI:** 10.4274/jtgga.2017.0027

**Published:** 2017-09-01

**Authors:** Fatma Beyazıt, Mesut A. Ünsal

**Affiliations:** 1 Department of Obstetrics and Gynecology, Çanakkale 18 Mart University Training and Research Hospital, Çanakkale, Turkey

To the Editor;

In the December 2016 issue of your journal, Fakor et al. (1) presented an original article entitled “The association between levels of maternal serum leptin in the third trimester and the occurrence of moderate preterm labor” in which the authors elucidated the possible role of leptin in the development of preterm labor in 30 moderate preterm delivering women. The authors demonstrated that low serum leptin levels may have a substantial predictive value for preterm birth before 34 weeks’ gestation by altering cytokine balance, cytotrophoblast and angiogenic activity, in the feto-placento-maternal unit. Although the authors presented and discussed their study results effectively in the context of previous studies, we believe that like any other study, there are some methodologic issues related to the present paper. In this context, from a methodologic point of view, we have several concerns including unmatched body mass index (BMI) values of study participants, and the presence of a probable insulin resistance (IR), which is associated with preterm birth and serum leptin levels.

First, as also stated by the authors, numerous factors are strictly related with serum leptin levels including obesity, insulin, glucocorticoids, and thyroid hormones via multiple signaling pathways (2). From these variables, obesity presents a unique importance while evaluating serum leptin levels because of the fact that obese subjects have higher serum leptin values, which correlates body weight percentage, than normal weight subjects (3). The importance of BMI on serum leptin levels was demonstrated in a study by Paul et al. (4). Serum leptin levels and differences between sexes were found to be significant in all body mass indices. In this context, when comparing two groups to evaluate serum leptin levels, it is crucial to match study groups in respect to BMI values. The significantly higher BMI levels of control subjects (p=0.017) could be the reason of elevated leptin levels. In order to rule out this bias, an additional statistical analysis is needed to understand if serum leptin levels are affected by the BMI values of study participants.

Second, although the authors stated that they excluded patients with diabetes mellitus, they gave no information about the possible presence of IR in their study group. IR is especially important for a study investigating a connection between leptin and preterm labor because IR contributes to the relatively wide variation in leptin levels, which is seen even at similar levels of body mass (5, 6). Moreover, IR below the thresholds of gestational diabetes mellitus (GDM) can cause adverse maternal and perinatal outcomes. In a recent large-scale retrospective study by Temming et al. (7), it was demonstrated that even in the absence of GDM, IR was associated with increased risks of preterm birth. Based on these data, it is reasonable to suggest that the alterations on serum leptin levels could be associated with IR and resulting preterm labor.

In conclusion, as we see an increasing number of obstetric complications including preterm labor, this study covers an important and interesting topic. We believe that the further understanding of the roles of adipocyte-derived hormones in human pregnancy will provide an insight into metabolic risks associated with preterm labor.
