# Supplementary material for: Obesity and insulin resistance are significant predictors of serum leptin levels
Source: J Turk Ger Gynecol Assoc. 2017 Sep 1;18(3):158–9. doi: 10.4274/jtgga.2017.0027 (PMC5590214; doi:10.4274/jtgga.2017.0027)
Supplement: Related texts

# Reply: Author's Response

**Authors:** Fereshteh Fakor, Seyedeh Hajar Sharami, Forozan Milani, Fariba Mirblouk, Sodabeh Kazemi, Davoud Pourmarzi, Hannan Ebrahimi, Seyedeh Fatemeh Dalil Heirati

**Affiliations:** 1 Reproductive Health Research Center, Department of Obstetrics and Gynecology, Guilan University of Medical Sciences, Rasht, Iran; 2 Reproductive Health Research Center, Department of Epidemiology, Guilan University of Medical Sciences, Rasht, Iran

Dear Editor;

In a letter entitled “Obesity and insulin resistance are significant predictors of serum leptin levels,” two main criticisms were mentioned. The first was the effect of body mass index (BMI) in leptin levels, which has been established in many previous studies (1, 2). We mentioned it in the introduction of the paper and also considered BMI as an important confounding variable in our study (3). As a basic study design concept, we tried to match BMI variables in the case and control groups. However, we could not select two BMI-matched groups (p=0.017) because of the lack of patients with preterm labor. Therefore, we decided to decrease the effect of the BMI variable by statistical methods. The other criticism was the effect of insulin resistance (IR) on leptin level. As it was written in methods section of our article (3), we tried not to include conditions related with IR such as diabetes and polycystic ovarian syndrome. When patients are hospitalized in Iran, their blood sugar is checked as a routine lab test. In addition, a glucose tolerance test is performed between 24-28 weeks of pregnancy as a screening program in Iran.

## References

[1] Paul RF, Hassan M, Nazar HS, Gillani S, Afzal N, Qayyum I (2011). Effect of body mass index on serum leptin levels. J Ayub Med Coll Abbottabad.

[2] Considine RV, Sinha MK, Heiman ML, Kriauciunas A, Stephens TW, Nyce MR, et al (1996). Serum immunoreactive-leptin concentrations in normal-weight and obese humans. N Engl J Med.

[3] Fakor F, Sharami SH, Milani F, Mirblouk F, Kazemi S, Pourmarzi D, et al (2016). The association between level of maternal serum leptin in the third trimester and the occurrence of moderate preterm labor. J Turk Ger Gynecol Assoc.
